# Comprehensive Assessment of Drought Tolerance in Native Melon Germplasms from Xinjiang at the Germination Stage

**DOI:** 10.3390/plants15152249

**Published:** 2026-07-23

**Authors:** Yafei Li, Qingqing Wang, Junzhuo Yang, Shan Su, Tiantian Yang, Huilin Wang, Zuyun Dai, Zhongzhou Yang, Feishi Luan, Shi Liu, Chong Du, Chaonan Wang

**Affiliations:** 1 College of Horticulture, Xinjiang Agricultural University, Urumqi 830052, China; 19590159038@163.com (Y.L.); 18366047073@163.com (Q.W.); yang260822@163.com (J.Y.); sushan0823@163.com (S.S.); yttneau2021@gmail.com (T.Y.); wanghuilin@126.com (H.W.); godv2018@163.com (C.D.); 2 Xinjiang Special Melon and Fruit Variety Improvement and Logistics Transportation Joint Research Center, Xinjiang Agricultural University, Urumqi 830052, China; 3 China-Costa Rica Belt and Road Joint Laboratory on Fruit and Vegetable Bio-Breeding and Intelligent Technology, Anhui Jianghuai Horticulture Technology Co., Ltd., Hefei 230031, China; daizuyun@jhseed.com; 4 Key Laboratory of Fruit and Vegetable Germplasm Innovation and Utilization in Jianghuai Region, Ministry of Agriculture and Rural Affairs, Anhui Jianghuai Horticulture Technology Co., Ltd., Hefei 230031, China; yangzhongzhou@jhseed.com; 5 College of Horticulture and Landscape Architecture, Northeast Agricultural University, Harbin 150030, China; luanfeishi@neau.edu.cn (F.L.); shiliu@neau.edu.cn (S.L.)

**Keywords:** melon, drought stress, drought tolerance screening, germplasm evaluation, physiological response

## Abstract

Drought and water deficit have severely restricted melon (*Cucumis melo* L.) production in Xinjiang, and large-scale systematic evaluations of drought tolerance at the germination stage are still extremely limited. Physiological and biochemical indicators related to the germination stage, including osmotic adjustment substances and antioxidant enzyme activities, have not yet been incorporated into prediction models for the rapid identification of germplasm drought resistance. To address these research gaps, this study selected 60 accessions of local melon germplasm resources in Xinjiang and used polyethylene glycol (PEG) solutions at four different concentrations (0%, 10%, 20% and 30%) to simulate drought stress conditions. Drought tolerance was evaluated to develop a method for the rapid screening of drought-tolerant germplasms. The findings demonstrated that PEG stress significantly suppressed seed germination and had both stimulatory and inhibitory effects on radicle growth. With the increase in PEG concentration, germination indices consistently exhibited a downward trend. Under 10% PEG treatment, the variation among different germplasms was relatively small, while 30% PEG completely inhibited seed germination. Notably, 20% PEG fell within the semi-lethal concentration range for all tested germplasms and yielded the maximum coefficient of variation for germination rate, which could maximally differentiate the drought resistance differences among germplasms. Therefore, 20% PEG was determined to be the optimal screening concentration. Under 20% polyethylene glycol (PEG) stress, the degree of membrane lipid peroxidation (malondialdehyde, MDA), contents of osmotic regulators (proline, Pro; soluble protein, SP), and activities of antioxidant enzymes (superoxide dismutase, SOD; peroxidase, POD; catalase, CAT; ascorbate peroxidase, APX) in the radicles of melon germplasms were universally elevated. However, the variation ranges and trends of biochemical indices among different germplasms exhibited significant differences. The proline content of melon accessions with strong drought resistance increased, the malondialdehyde (a product of membrane damage) was low, and the enzyme activities increased significantly. The proline content of non-drought-tolerant melon accessions increased less, malondialdehyde accumulated in large amounts, and the activity of some protective enzymes decreased. Correlation analysis demonstrated that Pro exerted a synergistic effect in conjunction with antioxidant enzymes (SOD, CAT) to mitigate drought stress. Cluster analysis classified the germplasm into 14 high-tolerance types, 10 medium-tolerance types, and 9 low-tolerance types. Based on extreme germination phenotypes, 27 germplasms were identified as drought-sensitive types. A prediction model for drought tolerance was established via stepwise regression: D = −0.309 + 0.053 × Pro (proline content) + 0.319 × RL (radicle length) + 0.469 × MDA (malondialdehyde) + 0.137 × SOD (superoxide dismutase), with four core indicators (RL, MDA, Pro, SOD) identified. These findings provide a scientific basis and technical support for drought tolerance breeding, parental selection, and large-scale, precise, and rapid drought tolerance screening of melon germplasms in the arid regions of Xinjiang.

## 1. Introduction

Melon (*Cucumis melo* L.) is a globally important horticultural crop [1]. China is the world’s top melon producer, with its cultivation area and output accounting for 36.23% and 49.09% of the global total, respectively [2]. However, the intensification of global climate change and the scarcity of water resources have rendered drought stress one of the primary abiotic stresses impacting the growth, development, yield formation, and fruit quality of horticultural crops [3,4]. As a representative arid region, Xinjiang has scarce precipitation and insufficient irrigation water resources. Drought and water scarcity often lead to a substantial decline in melon production [5]. Consequently, screening and identifying drought-resistant varieties are crucial measures for the development of stress-resistant crops and the guarantee of agricultural production [6].

Seed germination is the most sensitive stage for plants in response to water deficit. The drought tolerance at this stage directly determines the field seedling emergence rate and is closely associated with the subsequent crop yield [7]. Drought is the primary stress factor restricting seed germination and plant growth. Inadequate water supply notably impedes seed metabolism and hinders the germination process [8]. As stress intensifies, the seed germination rate, germination potential, germination index, and vigor index all exhibit a significant decline [9].

In response to drought stress, plants activate a series of drought-tolerance response mechanisms. The elevation of malondialdehyde (MDA) content acts as a crucial indicator of oxidative stress induced by drought. As a product of membrane lipid peroxidation, it can be used to assess the degree of cell membrane damage and plant drought tolerance [10,11]. In the context of osmotic regulation, proline (Pro) and soluble protein (SP) act as crucial osmotic regulatory substances. Their contents gradually increase with the intensification of drought stress, which helps maintain cell osmotic pressure and mitigate cellular damage [12,13]. Plants rely on their antioxidant system to counteract drought-induced oxidative damage. This system primarily consists of superoxide dismutase (SOD), peroxidase (POD), ascorbate peroxidase (APX), and catalase (CAT) [14]. Drought leads to a substantial accumulation of reactive oxygen species (ROS) and impairs the activity of various enzymes in plants [15]. Meanwhile, the activities of enzymes such as CAT, APX, SOD, and POD typically increase significantly to scavenge excessive reactive oxygen species (ROS) and mitigate their harmful effects on cell membranes, chloroplasts, and enzyme structures, thereby enhancing drought tolerance [4,16].

Polyethylene glycol (PEG-6000) has been extensively used in the screening of drought-resistant crop germplasms due to its stable stress-inducing effect and high controllability [17]. Based on this method, previous researchers have conducted drought-resistant germplasm screening in multiple crop species. For example, Ahmed et al. [18] identified the drought tolerance of 80 different wheat genotypes and selected wheat lines with strong drought tolerance, such as NR-499, NARC-2009, and Pakistan-2013. Similar methods have been applied to the screening of drought-resistant lines of *Cucumis sativus* L. Reference [19] and *Zea mays* L. [20]. Current research on melon drought resistance primarily focuses on physiological responses and drought-resistance gene mining at the seedling stage, and there are still notable gaps in the systematic drought-resistance screening of melon germplasm resources [21,22]. Although Xinjiang has abundant region-specific indigenous melon germplasm resources, a large-scale systematic identification of drought resistance during the germination stage for these local germplasms has not been carried out to date. Existing studies on the drought resistance evaluation of melon germplasm at the germination stage mostly use a single phenotypic trait, such as germination rate, as the evaluation basis and lack a multi-dimensional comprehensive evaluation system that integrates germination growth indicators, osmotic adjustment substances, and antioxidant enzyme activities [2]. Furthermore, few studies have integrated relevant physiological and biochemical indicators at the germination stage to construct drought resistance prediction models, making it difficult to achieve rapid drought resistance grading for large batches of germplasm. In view of these limitations, this study selected 60 native melon germplasm accessions from Xinjiang to screen for local germplasm resources with high drought tolerance and integrated germination morphological and physiological indicators to construct a drought resistance prediction model, aiming to fill the gap in the rapid drought resistance evaluation system for Xinjiang melon germplasm at the germination stage.

Traditional evaluations of plant drought tolerance are predominantly based on a single germination trait, such as germination rate (GR). Although this approach can reveal phenotypic differences among different germplasms, it cannot fully characterize the complex physiological and metabolic regulatory networks in plants under drought stress. Evaluation based on a single indicator is also highly vulnerable to environmental fluctuations, which often leads to biased assessment results [2,23]. In this study, the multi-index membership function method was used to integrate multiple types of indicators, including germination traits, osmotic adjustment substances, and antioxidant enzyme activities, into a comprehensive evaluation system. The comprehensive evaluation value D was calculated based on the coefficient of variation for each indicator, which effectively reduces the deviation caused by single-indicator evaluation and enables a more objective assessment of the overall drought tolerance of the tested germplasms. On this basis, core indicators were further screened through stepwise regression analysis, and a corresponding prediction model was constructed. This method simplifies the measurement process while guaranteeing evaluation accuracy and provides technical support for rapid drought tolerance screening of large-scale germplasm resources.

In this study, 60 local melon germplasm resources from Xinjiang were used as experimental materials. Polyethylene glycol 6000 (PEG-6000) was employed to simulate drought stress, and drought tolerance at the germination stage was evaluated to screen for highly drought-tolerant germplasms. A rapid drought tolerance screening model was established, and key physiological and biochemical indicators for drought tolerance prediction were selected. This research provides a scientific basis and a technical approach for drought tolerance breeding, elite parent selection, and large-scale, accurate, and rapid drought tolerance screening of melon germplasms in the arid regions of Xinjiang.

## 2. Results

### 2.1. Effects of Different Concentrations of PEG on Germination Rate, Germination Potential, and Radicle Length of Melon Seeds

As the PEG concentration increased, both the germination rate (GR) and germination potential (GP) of melon seeds gradually decreased, while the degree of variation continued to rise (Table 1). Under the control (CK) condition, the average GR and GP were 83.19% and 82.75% respectively, with coefficients of variation (CV) of 0.29 and 0.30, indicating a low level of genotypic differentiation. At a 10% PEG concentration, the GR and GP decreased to 75.53% and 74.44% respectively, while the CV for both parameters increased to 0.37, suggesting notable inter-population variations under drought stress. At 20% PEG, the GR and GP further declined to 56.53% and 54.13% respectively, with CV values of 0.60 and 0.62, indicating substantial genotypic divergence in drought tolerance. When exposed to 30% PEG, both the GR and GP dropped to zero, meaning that germination was completely suppressed in all tested germplasms.

The effects of different PEG concentrations on radicle length (RL) are presented in Figure S1 and Table 1. Under the control (CK) condition, the mean RL was 8.11 cm (CV = 0.58), reflecting inherent variation in radicle growth across germplasms. The CV under the 10% PEG treatment was higher than that under CK. PEG exerted both stimulatory and inhibitory effects on radicle elongation, showing genotypic differences in stress responses. At 20% PEG, RL significantly decreased to 1.02 cm, while the coefficient of variation (CV) increased to 1.04, indicating severe inhibition of root growth and substantial genotypic variation. A 30% PEG concentration exceeded the tolerance threshold for seed germination and radicle development in all tested melon germplasms, leading to the complete inhibition of relevant physiological processes.

### 2.2. PEG Concentration Screening Map for Identifying Drought Tolerance of Melon at the Germination Stage

Linear regression analysis was conducted to examine the relationship between PEG stress concentration (independent variable, *x*) and relative germination rate (dependent variable, *y*) for each melon germplasm (Table S2). The semi-lethal concentration, defined as the PEG concentration at which the relative germination rate of seeds decreases to 50%, can objectively reflect the critical drought tolerance threshold for each germplasm. The coefficient of variation for germination rate can quantify the degree of differentiation in drought response among all tested germplasms. Accordingly, these two indicators were jointly adopted as the screening and evaluation criteria in this study.

The semi-lethal concentration of the 60 tested melon germplasms ranged from 10.17% to 24.81%, and 20% PEG fell exactly within the critical concentration range of the vast majority of experimental materials. Meanwhile, the inter-germplasm coefficient of variation for germination rate under 20% PEG treatment reached its peak, which generated the maximum phenotypic difference and enabled clear differentiation between drought-sensitive and drought-tolerant germplasms. In contrast, the low concentration 10% PEG provided insufficient stress intensity, resulting in negligible differences in drought tolerance performance among germplasms, while 30% PEG exerted excessively strong inhibition, making it impossible to identify the drought tolerance variation in moderately drought-tolerant germplasms. In conclusion, a 20% PEG solution was ultimately determined to be the optimal stress concentration for the drought tolerance screening of melon germplasms at the germination stage in this study.

### 2.3. Effects of 20% PEG on Membrane Lipid Peroxidation and Osmotic Adjustment Substances in Radicle of Melon Seeds

Under 20% PEG stress, 27 melon germplasms showed a low germination rate, with radicle growth of less than 0.3 cm, which prevented them from completing the determination of biochemical indices. Therefore, the relevant biochemical indices of the remaining 33 germplasms were determined and analyzed.

**Figure 1:**
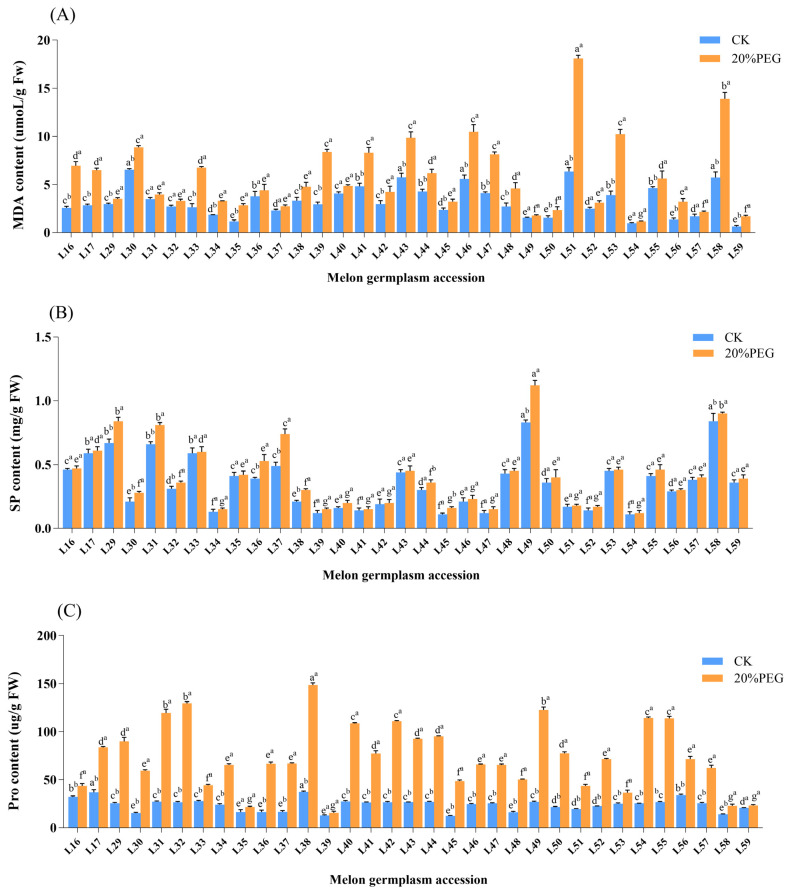
Influence of 20% PEG on the osmotic adjustment of melon seed radicles: (**A**) Influence of 20% PEG on the MDA content of melon seed radicles; (**B**) Influence of 20% PEG on the SP content of melon seed radicles; (**C**) Influence of 20% PEG on the Pro content of melon seed radicles. Note: Based on Duncan’s test (*p* ≤ 0.05), the unmarked lowercase letters signify significant differences among different melon varieties under the same treatment conditions, while the superscript letters denote significant differences among different treatments for the same melon germplasm. The histogram and the error bar represent the mean ± standard deviation.

Under 20% PEG-induced drought stress, the contents of malondialdehyde (MDA), proline (Pro), and soluble protein (SP) in the radicles of all tested melon germplasm accessions increased to varying degrees (Figure 1). The MDA contents of accessions L16, L17, and L39 increased significantly, with increments exceeding 100%, while much smaller rises were observed in L29, L36, L48, and L57 (Figure 1A). Overall, the soluble SP concentrations in melon radicles showed a slight upward trend. The SP levels of L29, L37, and L49 were significantly higher than those of the control group (CK), with L37 exhibiting the maximum increment of 51.02%. In contrast, the SP increments of L33, L43, and L48 were less than 5% and statistically insignificant (Figure 1B). The 20% PEG treatment significantly elevated the proline contents in most melon germplasm accessions. L32 showed the most dramatic proline increase of 388.78%, whereas only a few accessions, including L39 and L59, had no significant difference in proline content compared to CK (Figure 1C).

### 2.4. Effects of 20% PEG on the Activity of Antioxidant Enzymes in Radicle of Melon Seeds

**Figure 2:**
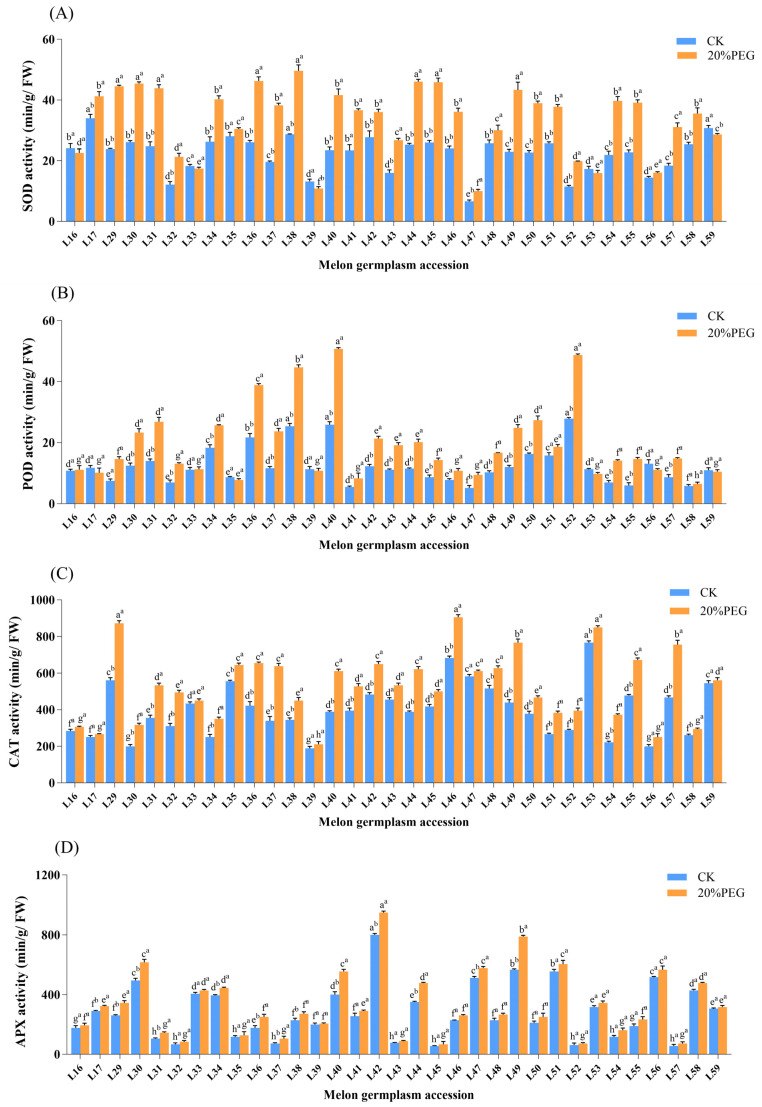
Influence of 20% PEG on the antioxidant enzyme activity of melon seed radicles: (**A**) Influence of 20% PEG on the SOD activity of melon seed radicles; (**B**) Influence of 20% PEG on the POD activity of melon seed radicles; (**C**) Influence of 20% PEG on the CAT activity of melon seed radicles; (**D**) Influence of 20% PEG on the APX activity of melon seed radicles. Note: Based on Duncan’s test (*p* ≤ 0.05), the unmarked lowercase letters signify significant differences among different melon varieties under the same treatment conditions, while the superscript letters denote significant differences among different treatments for the same melon germplasm. The histogram and the error bar represent the mean ± standard deviation.

After seven days of 20% PEG-induced drought stress treatment, the activities of four antioxidant enzymes in the radicles of all tested melon germplasm accessions were altered. SOD and POD activities increased in most accessions but decreased in a small subset, whereas CAT and APX activities increased in all tested germplasms (Figure 2). Figure 2A shows that the superoxide dismutase (SOD) activities of L29, L37, L49 and other lines were significantly increased. Among them, L37 exhibited the highest increment, with an increase of 95.25% compared to the control (CK). In contrast, SOD activities declined to varying degrees in L16, L33, L59 and other materials, among which L39 had the maximum reduction of 17.68%. Measurements of POD activity revealed that L55 possessed the most prominent increase (144.45%), and significant elevations were also observed in L29, L52, and L57. Nevertheless, POD activities decreased under drought stress in L17, L35, and L59, with L17 showing the largest reduction of 14.08% (Figure 2B). Figure 2C illustrates markedly enhanced CAT activities in L36, L40, L54 and other accessions. L37 achieved the highest CAT increment (88.52%), while relatively minor increases were detected in L33 and L59. APX activities of L37 and L54 increased by 46.15% and 38.10%, respectively. By comparison, the increments for L39 and L59 were only 2.78% and 3.64% (Figure 2D).

### 2.5. Correlation Analysis of Physiological and Biochemical Indexes of the Radicle of Melon Seeds

**Figure 3:**
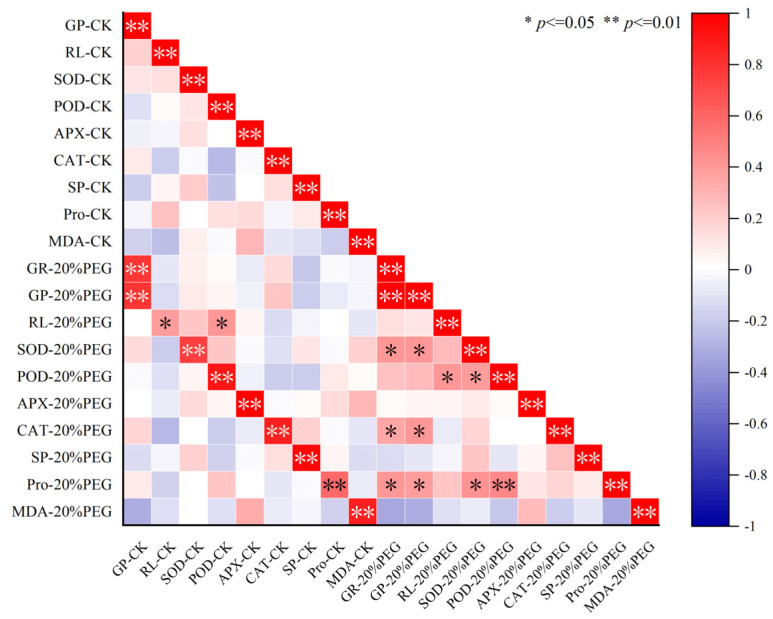
Correlation analysis between seed germination indices and physiological and biochemical indices. Note: Red and blue denote positive and negative correlations, respectively. The asterisks (*, **) represent the significance levels of 0.05 and 0.01, respectively.

To elucidate the correlations among the indexes under CK and 20% PEG stress conditions, Pearson correlation analysis was employed to examine the relationships between germination and physiological and biochemical indexes under the CK and 20% PEG treatments (Figure 3). Under the CK condition, only the GR exhibited a significant correlation with GP, while no significant correlations were observed among other indexes. Under 20% PEG stress, germination capacity indicators (GR, GP) showed significant positive correlations with Pro content, SOD activity and CAT activity, respectively.

Pro, SOD and POD were positively correlated with one another. The correlations between Pro and SOD, as well as between SOD and POD, were significant, whereas the correlation between Pro and POD was highly significant. In addition, RL under 20% PEG stress showed a significant positive correlation with POD activity and root length under the CK.

### 2.6. Evaluation of Drought Tolerance of Melon Germplasm Resources at the Germination Stage

**Figure 4:**
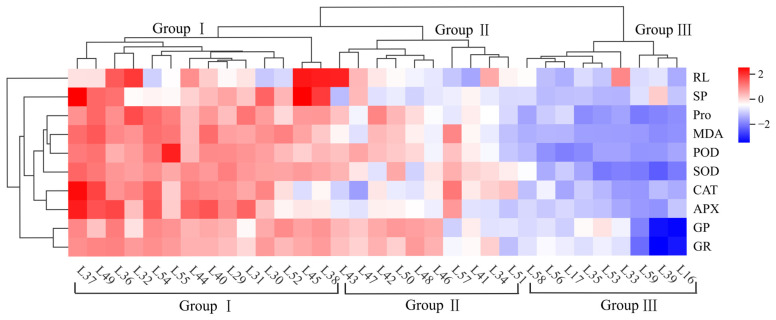
Tree diagram and heat map analysis of 33 melon germplasms subjected to drought stress.

In this study, the main objective was to screen germplasms with high drought tolerance. Under 20% PEG stress, the germination and root growth of 27 melon germplasms were significantly inhibited, making it impossible to determine their biochemical indices. Considering practical production, these germplasms exhibited suboptimal seedling emergence capacity under drought conditions. Based on the phenotypic traits at the germination stage, they were identified as drought-sensitive types, accounting for 45.00% of the total tested melon materials (Table 2). The membership function method was used to calculate and rank the comprehensive drought tolerance evaluation value D for the remaining 33 melon germplasms (Table 3). Cluster analysis was then conducted using the membership function values of 10 indicators. Based on the membership function values of germination phenotypes, radicle lengths and various physiological and biochemical indexes of 33 germplasms under 20% PEG stress, the Ward deviation square sum method and Euclidean distance were used to carry out systematic cluster analysis. Combined with the Euclidean distance segmentation node of the clustering tree, the 33 germplasms were divided into three drought tolerance types. As shown in Figure 4, these 33 drought-resistant melon germplasm resources were classified into three groups: high-tolerance, moderate-resistance, and low-tolerance types.

The first group, accounting for 23.33% (14 varieties) of the total melon germplasm resources, showed high drought tolerance with a relatively high D value (average 0.67, Table 2). Moreover, the membership function values were high for almost all indicators. The second group, exhibiting moderate drought tolerance, accounted for 16.67% (10 varieties) of the total tested germplasm resources, with an average D value of 0.48. The membership function values of this group for most indicators were slightly lower, indicating that it could tolerate low-intensity drought. Group III, consisting of 9 varieties, was classified as mildly drought-sensitive, with an average D value of 0.15, accounting for 15.00% of the total tested melon materials.

### 2.7. Establishment of a Regression Model for the Drought Tolerance of Melons

A stepwise regression approach was used to develop a prediction model for melon drought tolerance during the germination stage. The comprehensive membership function D value was designated as the dependent variable, while the positive drought-tolerance coefficients of each germination-stage index were regarded as independent variables. In the stepwise regression procedure, four stepwise regression models were constructed by incorporating independent variables based on their contributions (Table 4).

Proline (Pro), radicle length (RL), malondialdehyde (MDA), and superoxide dismutase (SOD) jointly characterize plant drought tolerance across four dimensions: osmotic regulation, root development, membrane damage, and antioxidant capacity. Germination rate (GR) was excluded from the analysis due to information redundancy with Pro and SOD; catalase (CAT) was eliminated because of collinearity with SOD; soluble protein (SP), peroxidase (POD), and ascorbate peroxidase (APX) were not retained, as their contributions to drought tolerance characterization were not statistically significant (*p* > 0.05). With the independent variables gradually incorporated into the model, the adjusted R^2^ value continued to rise, the overall F test results of each model reached a very significant level, and the model fitting degree was improved. Model 4 with the highest adjusted R^2^ was selected as the optimal model, with an adjusted R^2^ of 0.989, indicating that this model could explain 98.9% of the total variance of the D value. Model 4 revealed that Pro, RL, MDA and SOD were the key factors affecting the drought resistance of melon seeds during germination. Therefore, under identical experimental conditions, this equation can be used to rapidly estimate the comprehensive subordinate function D value for drought resistance identification, making it suitable for large-scale screening of melon germplasm accessions.

## 3. Discussion

A concentration of 20% PEG was identified as the screening concentration, which was consistent with the stress concentration adopted by Wu et al. [5] for drought-resistant cultivar screening of Xinjiang Hami melon. The germination rate, germination potential and radicle length of cucurbit seeds gradually decreased with increasing PEG concentration, which was in line with previous studies on *Cucumis sativus* L. [24], *Glycine max* L. Merr. [25] *Brassica napus* L. [26], *Zea mays* L. Reference [27] and Cucurbitaceae [28]. It is worth noting that the radicle length of some melon germplasms, such as L36 in this study, increased first and then decreased with the increasing PEG concentration, which was consistent with the conclusion of Taminždić et al. [29] in the drought stress test of cucumbers with different genotypes. Similar phenomena were also observed in *Zea mays* L. Reference [30] and *Lens culinaris* Medik. [31]. This suggests that under moderate stress, the plant defense system may be more effectively stimulated, ultimately enabling the plant to grow better under drought stress conditions [32]. When plants perceive water deficit, this low-concentration-induced root elongation phenomenon may also occur: plants activate the root osmotic regulation system and actively maintain the division capacity of root tip cells, thereby promoting water absorption from deeper soil layers [33]. Melon seed germination was completely inhibited under 30% PEG stress, yet some halophytes and woody species can still maintain a certain level of germination activity under the same intensity of osmotic stress [34,35]. In contrast, melon, as a typical horticultural crop, is more sensitive to water deficit during seed germination.

Cluster analysis demonstrated that drought-tolerant germplasms exhibited higher osmotic adjustment capabilities and antioxidant enzyme activities. As documented in prior research, plants accumulate osmolytes (e.g., SP, Pro) and enhance the activities of antioxidant enzymes such as SOD, CAT, APX, and POD in response to drought stress [36,37], which is consistent with the results of this study. Cucumber [23], watermelon [38] and other cucurbit crops [28] also showed similar physiological response characteristics, all of which reduced oxidative damage caused by drought by increasing osmotic adjustment and various antioxidant enzyme activities. The results of the correlation analysis indicate that antioxidant indicators including Pro, SOD and CAT show significant positive correlations under drought stress. However, these results only reflect correlational relationships at the numerical level and cannot confirm the existence of direct regulatory causal associations between these indicators. It is hypothesized that proline and the two antioxidant enzymes mitigate oxidative damage and osmotic stress induced by drought via synergistic responses. The plant drought response is a complex biological process regulated by multiple pathways, and plant drought resistance relies on the synergistic action of multiple functional substances. An increase in the activity of a single enzyme may not lead to a significant improvement in overall drought tolerance [39]. The aforementioned physiological indicators merely reflect response characteristics under drought stress, while the upstream and downstream molecular regulatory mechanisms underlying these responses still require further validation through subsequent experimental investigation. In this investigation, the activities of POD and SOD in certain drought-sensitive melon germplasms, such as L35 and L53, decreased after 7 d of stress. This phenomenon can be ascribed to the imbalance of the antioxidant system and excessive accumulation of reactive oxygen species in cucurbit crops under severe or long-term drought conditions [40]. The bidirectional alteration of enzyme activity resulting from this genotypic difference is not an anomaly. Some studies have exposed three different melon genotypes to PEG stress, finding that SOD and POD activities in sensitive varieties continued to decline, while those in drought-tolerant varieties increased steadily [41]. Antioxidant enzyme activities in pumpkin [42], melon [43], and watermelon [38] showed varying degrees of increase or decrease under drought stress. In contrast, antioxidant enzymes in most gramineous crops typically exhibit a unidirectional increase under drought conditions, with only a few instances of decrease [44]. This phenomenon implies the diversity and genotype-specific nature of drought tolerance and antioxidant mechanisms in cucurbit crops.

Some studies have found that after drought stress, drought-tolerant melon germplasms accumulate less MDA, which is consistent with the results of this study [1]. Owing to the higher efficiency of the antioxidant and osmotic regulation systems in drought-tolerant varieties, they generally exhibit lower MDA content [11]. Their content can be used to assess the degree of cell membrane damage and plant drought tolerance [10,11]. Similar studies have shown that MDA content in drought-resistant wheat [45] and melon cucumber [39] increased less. Under osmotic stress induced by 20% PEG, the increase in MDA content in germplasm with high drought tolerance was significantly lower than that in drought-sensitive germplasm. As MDA is the final product of membrane lipid peroxidation, these results indicate that highly drought-tolerant germplasm may possess a more stable membrane lipid composition or stronger membrane repair capacity under osmotic stress [46]. After 7 d of 20% PEG stress, the SP and Pro contents of melon increased, which was consistent with the response trends of *Citrullus lanatus*, *Glycine max* (L.) Merr. [13], and *Solanum lycopersicum* L. Reference [12] under drought stress. In our study, the increment of proline content under 20% polyethylene glycol stress was greater than that of other biochemical indicators. This may be because plants synthesize abundant proline to maintain normal osmotic adjustment under water deficit conditions [35]. Previous studies have demonstrated that plants can alleviate membrane damage by accumulating proline [40], while exogenous proline can boost the activities of antioxidant enzymes and mitigate oxidative injury [47]. These findings are consistent with the stress responses observed in wheat [48] and celery [49]: proline plays a vital role in reactive oxygen species detoxification, preservation of membrane integrity and stabilization of multiple enzyme activities. Accordingly, elevated proline levels can strengthen drought tolerance in plants [50]. Under osmotic stress, the average increase in proline content was significantly higher than that of soluble protein. Specifically, proline content in some germplasm materials reached as high as 388%. This result indicates that melon tends to adopt the proline biosynthesis pathway as the main osmotic adjustment strategy, rather than relying on the accumulation of soluble protein [5,46].

In this study, the germination rate and root growth of 27 melon germplasm accessions were severely inhibited under 20% polyethylene glycol (PEG) stress. Based on such extreme germination phenotypes, these accessions were directly classified as extremely drought-sensitive genotypes. This evaluation method has precedent in research on other crops. For instance, Yadav et al. [51] identified the tomato cultivar Srijana as drought-sensitive since it failed to germinate completely under PEG stress. Mannan and Begum [52] adopted a similar approach to assess germination phenotypes of 97 maize germplasm accessions and categorized those with poor performance as drought-sensitive. A multi-index comprehensive evaluation method was applied to the remaining 33 accessions available for biochemical determination. Moreover, a single indicator cannot fully reflect drought tolerance, whereas comprehensive evaluation with multiple indicators can markedly improve the reliability of evaluation results [2,23]. The 27 melon germplasm resources with low germination rates and radicle lengths of less than 0.3 cm were classified as drought-sensitive types. This threshold was determined based on measured data from melon germination experiments under 20% PEG-induced drought stress. Under this stress condition, only a small number of seeds can germinate, and the longest radicle among germinated seeds was only 0.3 cm. Seedling radicles of this length do not have sufficient fresh weight, making it impossible to obtain complete data for all physiological and biochemical indicators. To standardize the sampling protocol and ensure the comparability of experimental data, 0.3 cm was selected as the critical classification threshold.

In our research, 33 melon germplasms were divided into three categories via clustering analysis of 10 indexes using membership function values. Similar methods have been applied to drought tolerance evaluation of 13 watermelons [38] and 58 melons [1]. Additionally, four key drought tolerance indexes (Pro, RL, MDA, SOD) for the melon germination stage were screened out through a drought tolerance prediction model constructed via stepwise regression. CAT and SOD showed collinearity and were preferentially eliminated during screening. The remaining unselected indexes had weak correlations with melon drought tolerance at the germination stage, failing to effectively improve model fitting accuracy and prediction ability, so they were excluded from the model. Compared with the prediction model constructed by Ren et al. [2] using data from 15 melon genotypes at the seedling stage, this study has a larger sample size and focuses on the germination stage—the most drought-sensitive stage for melons and a key stage determining seedling growth in production. Consequently, the model is more closely aligned with actual production scenarios in arid regions and has higher reliability and application value. In this study, stepwise regression was used to screen the core indicators of drought resistance, which could eliminate the multicollinearity of indicators and adapt to multi-index joint modeling. However, this method is limited by the dataset and lacks generalization, making it necessary to expand the germplasm samples to verify the stability of the results. It should be emphasized that the established model is only applicable to the preliminary screening of drought-tolerant melon germplasm during the seed germination stage, and its application still has certain limitations. In field cultivation, melons grown in Xinjiang face the risk of drought stress throughout the entire growth period.

In this study, 27 accessions were excluded from the modeling dataset because their poor germination under 20% PEG stress prevented the collection of sufficient radicle tissue for physiological assays. Given that model construction requires complete empirically measured datasets, forced inclusion of these incomplete records would inevitably introduce statistical bias. Conversely, modeling based exclusively on complete-case data enhances the reliability and robustness of the predictions. Accordingly, the established model is specifically applicable to the preliminary large-scale screening of germplasms with normal germination capacity. It is important to note that the model is confined to the Xinjiang local melon germplasm resources evaluated in this study and is limited to the germination stage; extrapolation to other germplasm populations or growth stages is not recommended. The results should be interpreted as a reference for subsequent refined identification rather than as definitive conclusions. For the 27 sensitive accessions that failed to pass the germination screening, their drought susceptibility has already been phenotypically confirmed. Should further physiological characterization be required, alternative assessments using lower concentrations of PEG-6000 at the seedling stage may be considered. Furthermore, in this study, both model construction and fitting performance evaluation were performed using the same dataset, and no independent external validation set was established. Although the adjusted coefficient of determination is as high as 0.989, this modeling strategy still carries a risk of overfitting. Specifically, while the model shows excellent adaptability to the germplasm data collected in this experiment, its prediction accuracy will likely decrease when applied to melon germplasms from other batches or other sources. Accordingly, future research needs to expand the independent sample dataset for external validation to further improve the generalizability of the model. Among the 60 Xinjiang local melon germplasm populations examined in this experiment, RL, Pro, MDA and SOD were the most informative indexes for drought resistance evaluation only at the germination stage. These indicators cannot be regarded as universal drought screening markers for all melon materials until further verification using independent germplasm populations from other geographical regions is completed.

Stepwise regression analysis was used to screen four core drought resistance indices, namely Pro, RL, MDA and SOD. Among these indices, root length is the only morphological index. During the germination stage, excellent root elongation ability can expand the water absorption range of seedlings and reduce the damage caused by drought stress, representing an intuitive morphological response of melon to early drought resistance [39]. Under field conditions, there may be a potential association between root length at the germination stage and drought resistance at the adult plant stage. Melon seedlings with outstanding root elongation ability under drought stress during germination have stronger early water uptake capacity and improved water absorption ability, which helps them accumulate growth advantages for subsequent development. This root-related advantage may persist into the adult stage, promoting the formation of deep root systems in the field and enhancing the long-term soil drought tolerance of plants. However, complex environmental factors in the field, such as soil structure, can change the growth trajectory of roots [53]. Therefore, root length at the germination stage and the drought resistance of adult plants may not be completely synchronized, and the relationship between the two remains unclear at present.

## 4. Materials and Methods

### 4.1. Experimental Materials and Experimental Site

This experiment was conducted in the Laboratory of the College of Horticulture, Xinjiang Agricultural University. A total of 60 melon germplasm resources were provided by the Watermelon and Melon Research Team of the College of Horticulture, Xinjiang Agricultural University. The names, numbers, and sources of these germplasms are listed in Table S1.

### 4.2. Experimental Design

Distilled water was set as the control (CK), and three concentrations (10%, 20% and 30%) of PEG-6000 (Macklin Biochemical Co., Ltd., Shanghai, China) were used to simulate drought stress for melon seed germination assays. Each treatment included 3 biological replicates, with 20 seeds per replicate. Melon seeds were immersed in 55 °C warm water for 15 min and then soaked in room-temperature water for 7 h. The treated seeds were evenly placed in 9 cm-diameter Petri dishes lined with double-layer filter paper, and 2.5 mL of the corresponding concentration of PEG solution was added to each dish. All the culture dishes were placed in an artificial climate incubator (Model DHP-9082D, Shanghai Qixin Scientific Instrument Co., Ltd., Shanghai, China) and incubated in the dark at 30 °C. Every 24 h, 2 mL of the corresponding concentration of PEG solution was added, and the filter paper was replaced every 48 h. Using the radicle breaking through the seed coat as the germination criterion, the germination index was measured from the second to the seventh day after the seeds were placed in the incubator. After 7 d of stress treatment, radicles were collected, immediately frozen in liquid nitrogen, and stored at −80 °C for subsequent physiological and biochemical analyses. The PEG concentration that most effectively differentiated the 60 melon germplasms was selected for the determination of physiological and biochemical indices. All common chemical reagents applied for physiological index measurement were purchased from Macklin Biochemical Co., Ltd., Shanghai, China.

### 4.3. Determination Indicators and Methods

The germination rate (GR) and germination potential (GP) were computed in accordance with the following formulas:

GR (%) = (the cumulative quantity of germinated seeds on the seventh day/the quantity of test seeds) × 100%

GP (%) = (the cumulative quantity of germinated seeds on the third day/the quantity of test seeds) × 100%

Radicle length (RL): On the 7th day after germination, root tip length was measured in centimeters (cm) using a ruler. The measured value was defined as the linear distance from the junction of the root base and hypocotyl to the tip of the root. Given the inherent differences in germination characteristics among different experimental germplasm materials, the number of germinated seeds varied across replicate groups. The statistical analysis procedure was performed as follows: first, the average root length of all germinated seeds in each replicate group was calculated, and the final results are presented as the mean of three independent biological replicates.

On the 7th day after germination, successfully germinated seeds with radicles protruding through the seed coat were collected for sampling (every 8 seeds per group, repeated three times). Radicles were cut off at the junction of the radicle and hypocotyl for subsequent determination of biochemical indicators. Three independent biological replicates were set up for biochemical indicator measurement, and the mean value and standard deviation were calculated based on the measurement results of the three replicates. Samples were ground in liquid nitrogen for subsequent measurements of malondialdehyde (MDA), proline (Pro), soluble protein (SP), superoxide dismutase (SOD), peroxidase (POD), catalase (CAT) and ascorbate peroxidase (APX). MDA content and the activities of SOD, POD and CAT were assayed following the protocol described by Huang et al. [54] Contents of SP and Pro, as well as APX activity, were determined using the method reported by Liu et al. [55].

### 4.4. Data Processing and Analysis

#### Drought Tolerance Coefficient and Membership Function Value

The calculation of the drought tolerance coefficient (DC) and membership value was performed according to the method proposed by Rehman et al. [1]. Depending on the different types of indicators, the corresponding calculation formulas were adopted, as presented below:

Positive indicators (GR, GP, Pro, SP, SOD, POD, CAT, APX): DC = stress measured value/CK measured value

Negative index (MDA): DC = CK measured value/stress measured value

Membership value = (X − X min)/(X max − X min)

In the formula, X represents the drought tolerance coefficient of a specific index for a given material, while X max and X min denote the maximum and minimum values of the drought tolerance coefficient for that specific index across all materials, respectively. The weight coefficient for each index in drought tolerance evaluation was determined based on the proportion of its coefficient of variation to the sum of the coefficients of variation for all indexes. Using these weight coefficients, the comprehensive membership function D value for each material was calculated.

### 4.5. Equation of Linear Regression

The mass fraction of PEG was designated as the independent variable (*X*-axis), while the relative germination rate of each melon germplasm under drought stress was defined as the dependent variable (*Y*-axis). Linear regression analysis was performed, and regression equations were established using Excel 2021. The semi-lethal concentration (the concentration at which the relative germination rate reaches 50%) for each melon germplasm accession was calculated using these regression equations.

### 4.6. Statistical Analysis

Data processing and preliminary statistics were completed using Microsoft Excel 2021, and all further statistical analyses were conducted using SPSS 27.0. One-way analysis of variance was performed to compare differences in multiple indicators across different melon germplasm resources under different PEG-6000 stress gradients, followed by Duncan’s new multiple range test for intergroup multiple comparisons. Pearson correlation analysis was used to investigate the correlations between indicators. Stepwise regression was adopted to screen core drought tolerance evaluation indicators, based on which a prediction model for drought tolerance during the melon germination stage was constructed. Prior to the one-way analysis of variance, Shapiro–Wilk test and Levene’s test were conducted to verify the normality and homogeneity of variance of the dataset. Data that did not conform to normal distribution were subjected to logarithmic transformation before subsequent statistical analysis. All figures and plots were generated using Origin 2022. Significance testing and regression model construction were performed via SPSS 27.0, while cluster analysis, correlation analysis, and figure plotting were completed with Origin 2022.

## 5. Conclusions

In this study, 60 local melon germplasms sourced from Xinjiang were subjected to PEG-6000 simulated drought stress to systematically screen and comprehensively evaluate their drought tolerance during the germination stage. A 20% polyethylene glycol (PEG) concentration was within the semi-lethal range for the tested germplasms. In conjunction with comparisons of the coefficients of variation for germination rate across various stress levels, 20% PEG was selected as the optimal concentration for drought tolerance screening. Drought stress significantly inhibited seed germination and radicle elongation. Germplasms with high drought tolerance exhibited superior osmotic adjustment capacities, heightened antioxidant enzyme activities, and less severe cell membrane damage. Correlation analysis indicated that osmotic adjustment (Pro) collaborated with antioxidant enzymes (SOD, CAT) to alleviate drought stress in melon germplasms under the 20% PEG treatment. The 60 germplasms were classified into four groups: highly tolerant, moderately tolerant, low-tolerant, and drought-sensitive. The 14 highly drought-tolerant germplasms identified can be employed as elite parental materials for drought tolerance breeding. A stepwise regression model, D = −0.309 + 0.053 × Pro + 0.319 × RL + 0.469 × MDA + 0.137 × SOD, was established to rapidly predict drought tolerance at the germination stage and improve the efficiency of large-scale drought tolerance screening in melon germplasms. In addition, in this study, model construction and fitting performance evaluation were performed using the same dataset, and no independent external validation set was established. Therefore, future research needs to expand the independent sample dataset for external verification to further improve the generalizability of the model. PEG-induced osmotic stress does not fully represent field drought conditions, only the germination stage was evaluated, and molecular validation of the selected physiological indicators was not performed.

## Figures and Tables

**Table 1:** Effects of different concentrations on germination rate, germination potential and radicle length of melon seeds.

Germination Index	PEG Concentration	Maximum Value	Minimum Value	Mean Value	Standard Deviation	Coefficient of Variation	Significance
GR/%	CK	100.00	15.00	83.19	24.18	0.29	<0.001
	10%	100.00	5.00	75.53	28.13	0.37	<0.001
	20%	100.00	0.00	56.53	33.64	0.60	<0.001
	30%	0.00	0.00	0.00	0.00	0.00	-
GP/%	CK	100.00	10.00	82.75	24.56	0.30	<0.001
	10%	100.00	5.00	74.44	27.84	0.37	<0.001
	20%	100.00	0.00	54.13	33.58	0.62	<0.001
	30%	0.00	0.00	0.00	0.00	0.00	-
RL/cm	CK	19.30	1.10	8.11	4.74	0.58	<0.001
	10%	13.40	0.20	3.96	3.16	0.78	<0.001
	20%	3.90	0.00	1.02	1.07	1.04	<0.001
	30%	0.00	0.00	0.00	0.00	0.00	-

Note: <0.001 indicates that the melon germplasm is extremely significant at the 0.05 significance level under the same treatment. -: Represents a null value. The same below.

**Table 2:** Grouping of 60 melon drought-resistant types.

Drought Tolerant Group	Tolerance Level	Average D Value	Percentage %
I	High-tolerance type	0.67	23.33%
II	Moderate resistance type	0.48	16.67%
III	Low-tolerance type	0.15	15.00%
IV	Sensitive drought type	-	45.00%

**Table 3:** Subordinate function values and drought-tolerance rankings based on the drought-tolerance coefficients of each index during the germination stage of melon seeds.

Number	GR	GP	RL	SP Content	Pro Content	SOD Activity	POD Activity	APX Activity	CAT Activity	MDA Content	D Value	Ranking
L16	0.4833	0.4167	0.0778	1.0385	1.3552	0.9325	1.0308	1.0937	1.0784	0.3551	0.05	33
L17	0.7833	0.7167	0.0873	1.0333	2.2732	1.2186	0.8592	1.1154	1.0667	0.4188	0.18	28
L29	0.9000	0.9000	0.3235	1.2580	3.5135	1.862	1.9556	1.3191	1.5545	0.7414	0.59	13
L30	0.9492	0.9153	0.1563	1.3514	3.8970	1.7384	1.8667	1.2472	1.5833	0.7708	0.56	14
L31	0.8636	0.8000	0.3784	1.2089	4.3899	1.7719	1.9167	1.3684	1.5000	0.7300	0.61	12
L32	0.9500	0.8333	0.8421	1.1304	4.8885	1.7550	1.8810	1.250	1.5893	0.7642	0.77	1
L33	0.7647	0.7692	0.6286	1.0135	1.6020	0.9539	1.0149	1.0548	1.0385	0.3767	0.29	25
L34	0.8750	0.7455	0.5370	1.0931	2.7241	1.5333	1.4000	1.1268	1.4000	0.5245	0.45	19
L35	0.7627	0.7966	0.2059	1.0360	1.3316	1.087	0.9038	1.0952	1.1600	0.3807	0.18	28
L36	0.9833	0.9833	0.7500	1.3326	4.0734	1.7804	1.7923	1.4063	1.5526	0.7872	0.77	2
L37	0.9667	0.9667	0.3846	1.4944	4.0505	1.9592	2.0286	1.4615	1.8852	0.838	0.74	4
L38	0.9474	0.9474	0.9167	1.4097	3.9779	1.7278	1.7632	1.1951	1.3065	0.6651	0.74	5
L39	0.4528	0.434	0.2308	1.1983	1.2338	0.8232	0.9559	1.0278	1.1176	0.3451	0.09	32
L40	0.9167	0.9167	0.4419	1.2256	3.9695	1.7736	1.9613	1.3889	1.5714	0.8384	0.67	7
L41	0.8167	0.8167	0.0638	1.1404	2.9367	1.5658	1.5152	1.1304	1.338	0.5786	0.35	23
L42	0.9167	0.9167	0.3684	1.0800	4.1846	1.3003	1.7297	1.1875	1.3448	0.6945	0.52	15
L43	0.8980	0.8980	0.9048	1.0250	3.4703	1.6745	1.7164	1.1429	1.1707	0.581	0.63	10
L44	0.9167	0.9000	0.6087	1.1919	3.5255	1.8221	1.7536	1.3651	1.6000	0.6888	0.65	8
L45	0.9286	0.9286	0.9310	1.4950	3.9614	1.7664	1.6538	1.2000	1.2000	0.7338	0.75	3
L46	0.9167	0.9167	0.2400	1.0885	2.6904	1.5055	1.3830	1.1463	1.3252	0.5334	0.38	22
L47	0.8727	0.8727	0.5000	1.2262	2.5855	1.5000	1.8387	1.1304	1.0476	0.5046	0.46	18
L48	0.9492	0.9322	0.2727	1.0582	3.1498	1.2433	1.6129	1.1707	1.2151	0.5910	0.41	21
L49	0.9800	0.8814	0.3889	1.3463	4.5605	1.8890	2.0694	1.3922	1.7468	0.8752	0.73	6
L50	0.875	0.9423	0.3188	1.0967	3.6012	1.7188	1.6735	1.1842	1.2353	0.6709	0.5	17
L51	0.7188	0.7500	0.3333	1.0703	2.2418	1.4690	1.1789	1.0900	1.4375	0.4433	0.32	24
L52	0.9074	0.9608	0.1983	1.2326	3.1811	1.7303	1.7485	1.1818	1.3654	0.7986	0.51	16
L53	0.7857	0.8302	0.1486	1.0163	1.4575	0.9231	1.0172	1.0877	1.1087	0.3791	0.16	30
L54	0.9649	0.9643	0.1765	1.1413	4.4976	1.8103	2.0238	1.3810	1.6750	0.8342	0.62	11
L55	0.9500	0.9500	0.3107	1.1338	4.2735	1.7191	2.4444	1.2353	1.4070	0.8189	0.64	9
L56	0.8000	0.7458	0.1269	1.0243	2.1002	1.1072	0.9306	1.0968	1.2500	0.4217	0.2	27
L57	0.7966	0.7193	0.1508	1.1002	2.4369	1.6924	1.7115	1.300	1.619	0.7898	0.44	20
L58	0.7679	0.7321	0.3134	1.0716	1.6225	1.3984	1.1143	1.1169	1.1277	0.4105	0.26	26
L59	0.6034	0.6034	0.2061	1.0785	1.1156	0.9303	0.9545	1.0364	1.0306	0.3694	0.11	31
coefficient of variation/%	15.49	16.86	67.16	11.74	37.09	22.41	27.31	10.11	17.06	28.85	-	-
Weight ratio/%	6.09	6.64	26.43	4.62	14.6	8.82	10.75	3.98	6.71	11.35	-	-

Note: SP: Soluble protein content; MDA: Malondialdehyde content; Pro: Proline content; POD: Peroxidase activity; SOD: Superoxide dismutase activity; CAT: Catalase activity; APX: Ascorbate peroxidase activity.

**Table 4:** Regression analysis between the D value and drought tolerance indexes.

Equation	Model	Adjust R^2^	F Test	Sig
(1)	D = −0.092 + 0.181 × Pro	0.856	179.938	<0.001
(2)	D = −0.128 + 0.161 × Pro + 0.276 × RL	0.929	197.612	<0.001
(3)	D = −0.249 + 0.058 × Pro + 0.350 × RL + 0.661 × MDA	0.980	489.648	<0.001
(4)	D = −0.309 + 0.053 × Pro + 0.319 × RL + 0.469 × MDA + 0.137 × SOD	0.989	680.064	<0.001

Note: Adjust R^2^ is the adjusted coefficient of determination; F test is the F test value of the overall significance of the model; Sig: at the 0.05 significant level; Pro is proline content; RL is radicle length; MDA is the content of malondialdehyde; SOD is superoxide dismutase activity.

## Data Availability

The original contributions presented in this study are included in the article. Further inquiries can be directed to the corresponding authors.

## Supplementary Materials

The following supporting information can be downloaded at: https://www.mdpi.com/article/10.3390/plants15152249/s1.

## Abbreviations

The following abbreviations are used in this manuscript:

GR	germination rate
GP	germination potential
RL	radicle length
MDA	malondialdehyde
Pro	proline
SP	soluble protein
SOD	superoxide dismutase
CAT	catalase
APX	ascorbate peroxidase
POD	peroxidase
PEG	polyethylene glycol
CV	coefficient of variation
DC	drought tolerance coefficient
CK	control
