# Supplementary material for: Comprehensive Assessment of Drought Tolerance in Native Melon Germplasms from Xinjiang at the Germination Stage
Source: Plants (Basel). 2026 Jul 23;15(15):2249. doi: 10.3390/plants15152249 (PMC13468599; doi:10.3390/plants15152249)
Supplement: Supplementary file 1 [file plants-15-02249-s001.zip › Supplementary Figure.pdf]

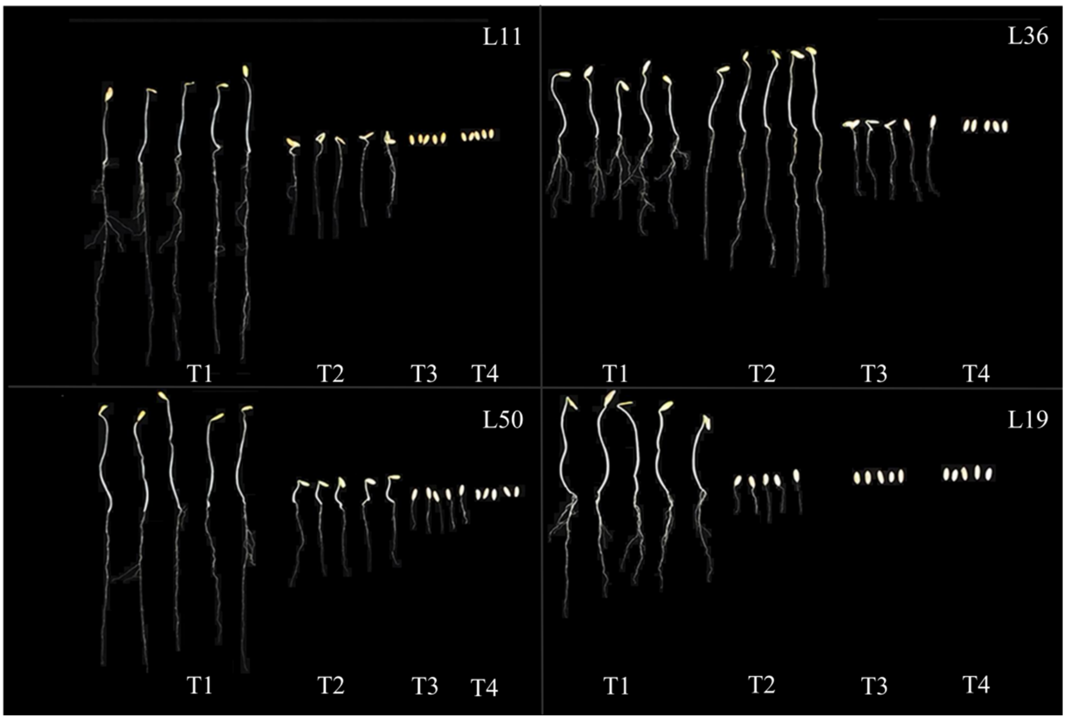

Figure S1 Influence of Varying PEG - 6000 Concentrations on the Root Length of Melon Seeds. Note: L11, L36, L50, L19 represent the numbers of melon germplasm. The scale is equivalent to 3 cm. In each region, T1: Control (CK), T2: 10% PEG, T3: 20% PEG, T4: 30% PEG.
