# Supplementary material for: Comprehensive Assessment of Drought Tolerance in Native Melon Germplasms from Xinjiang at the Germination Stage
Source: Plants (Basel). 2026 Jul 23;15(15):2249. doi: 10.3390/plants15152249 (PMC13468599; doi:10.3390/plants15152249)
Supplement: Supplementary file 1 [file plants-15-02249-s001.zip › Supplementary Table.pdf]

Supplementary Table S1 Information and source of 60 melon germplasms

Number	Germplasm Name	Source	Number	Germplasm Name	Source	Number	Germplasm Name	Source
L1	K-29 yellow skin white meat	Xinjiang, Kashgar	L21	Cerrekeqiligan	Xinjiang, Kashgar	L41	Kizbuya Coca-Cola	Xinjiang, Kashgar
L2	Quikebaier	Xinjiang, Kashgar	L22	Hamanchi	Xinjiang, Kashgar	L42	Bergzad	Xinjiang, Kashgar
L3	Baixiekexin	Xinjiang, Kashgar	L23	Popster leather teeth	Xinjiang, Kashgar	L43	Akpti No. 2	Xinjiang, Kashgar
L4	Yuan Baixiekexin	Xinjiang, Kashgar	L24	Red meat nameless	Xinjiang, Kashgar	L44	Green skin green meat	Xinjiang, Kashgar
L5	Salyk Aragher	Xinjiang, Kashgar	L25	Kukekkouchi	Xinjiang, Kashgar	L45	Postipiazi	Xinjiang, Kashgar
L6	Siklsu	Xinjiang, Kashgar	L26	Akpti No. 1	Xinjiang, Kashgar	L46	Aknawati 2	Xinjiang, Kashgar
L7	Aknawati 1	Xinjiang, Kashgar	L27	Single plant iron melon	Xinjiang, Kashgar	L47	Sheksin	Xinjiang, Kashgar
L8	Naxigan No.1	Xinjiang, Kashgar	L28	Circle Bischeksin	Xinjiang, Kashgar	L48	Potts tasty	Xinjiang, Kashgar
L9	Malay Tikazi	Xinjiang, Kashgar	L29	Uzbek	Xinjiang, Kashgar	L49	Green meat Jiashi melon	Xinjiang, Kashgar
L10	Quikbay heat	Xinjiang, Kashgar	L30	Shihezi Akeqi	Xinjiang, Kashgar	L50	Black skin green meat	Xinjiang, Kashgar
L11	Kalkasi	Xinjiang, Kashgar	L31	Iron melon	Xinjiang, Kashgar	L51	Yellow-skinned melon	Xinjiang, Kashgar
L12	Pishangua	Xinjiang, Kashgar	L32	Its power is dry	Xinjiang, Kashgar	L52	Mamitikazi	Xinjiang, Kashgar
L13	Artillery melon No.1	Xinjiang, Kashgar	L33	Local melon	Xinjiang, Kashgar	L53	Jiashi melon	Xinjiang, Kashgar
L14	Panakpoz is delicious	Xinjiang, Kashgar	L34	Naxigan 2	Xinjiang, Kashgar	L54	Kabyshekshin	Xinjiang, Kashgar
L15	Red meat Sheksin	Xinjiang, Kashgar	L35	Yingji Shawu Hot Airy	Xinjiang, Kashgar	L55	Jiaozi No.2	Xinjiang, Kashgar
L16	Akpti No. 1	Xinjiang, Kashgar	L36	Akekoukeqi No. 1	Xinjiang, Kashgar	L56	Cookbayge	Xinjiang, Kashgar
L17	Ketuo Kekouqi	Xinjiang, Kashgar	L37	Akekoukeqi selfing	Xinjiang, Kashgar	L57	Huang Kekou Keqi	Xinjiang, Kashgar
L18	Jiaozi No. 1	Xinjiang, Kashgar	L38	Akekoukeqi No.2	Xinjiang, Kashgar	L58	Kokki Yurik	Xinjiang, Kashgar
L19	Yellow net Wen Jiashi melon	Xinjiang, Kashgar	L39	Yellow skin white meat	Xinjiang, Kashgar	L59	My hot Aili	Xinjiang, Kashgar
L20	Shihezi White Akqi	Xinjiang, Kashgar	L40	Artillery melon No.2	Xinjiang, Kashgar	L60	Wure Aili mixed species	Xinjiang, Kashgar

Supplementary Table S2 Tolerant PEG concentration and PEG semi-lethal concentration of melon under drought stress

Number	Linear regression equation	Tolerance to PEG semi-lethal concentration	Number	Linear regression equation	Tolerance to PEG semi-lethal concentration
L49	y=-3.1600x+128.40<br>R^2=0.5565	24.81	L22	y=-3.2000x+114.67<br>R^2=0.8325	20.21
L31	y=-3.4545x+131.36<br>R^2=0.6265	23.55	L53	y=-3.0357x+110.71<br>R^2=0.7773	20.00
L36	y=-3.0167x+119.83<br>R^2=0.6133	23.15	L7	y=-3.3000x+115.33<br>R^2=0.8438	19.80
L54	y=-2.9474x+117.89<br>R^2=0.5985	23.03	L28	y=-3.233x+113.50<br>R^2=0.8469	19.64
L30	y=-3.0424x+120.00<br>R^2=0.6400	23.01	L4	y=-3.2373x+113.39<br>R^2=0.8505	19.58
L37	y=-3.0330x+119.67<br>R^2=0.6266	22.97	L8	y=-3.3833x+115.33<br>R^2=0.8691	19.31
L38	y=-3.0877x+120.88<br>R^2=0.6397	22.96	L33	y=-2.9412x+105.88<br>R^2=0.7764	19.00
L32	y=-3.0205x+119.33<br>R^2=0.6402	22.92	L20	y=-3.6757x+117.30<br>R^2=0.8579	18.31
L55	y=-3.0500x+119.50<br>R^2=0.6399	22.79	L59	y=-3.1897x+107.76<br>R^2=0.9128	18.11
L45	y=-3.0893x+120.00<br>R^2=0.6555	22.66	L16	y=-3.4167x+110.83<br>R^2=0.9361	17.8
L48	y=-3.0000x+117.46<br>R^2=0.6413	22.49	L9	y=-3.4621x+111.21<br>R^2=0.9346	17.68
L52	y=-2.9074x+115.37<br>R^2=0.6791	22.48	L27	y=-3.7500x+115.63<br>R^2=0.8612	17.50
L43	y=-3.1429x+120.61<br>R^2=0.6761	22.47	L11	y=-3.8108x+115.95<br>R^2=0.8447	17.31
L42	y=-3.0833x+119.17<br>R^2=0.6662	22.43	L39	y=-3.3962x+108.49<br>R^2=0.9591	17.22
L40	y=-3.0667x+118.50<br>R^2=0.6672	22.34	L13	y=-3.8864x+116.82<br>R^2=0.8192	17.19
L44	y=-3.0667x+118.50<br>R^2=0.6672	22.34	L5	y=-3.5254x+110.51<br>R^2=0.9397	17.16
L29	y=-3.1x+119.00<br>R^2=0.6792	22.26	L15	y=-3.7879x+112.12<br>R^2=0.8731	16.40
L50	y=-3.1607x+120.18<br>R^2=0.6935	22.20	L21	y=-3.9286x+114.29<br>R^2=0.8187	16.36
L41	y=-3.0197x+117.00<br>R^2=0.7475	22.19	L25	y=-3.2182x+100.55<br>R2=0.9847	15.71
L34	y=-3.1429x+119.46<br>R^2=0.6961	22.10	L19	y=-4.075x+113.00<br>R^2=0.7693	15.46
L46	y=-3.0167x+116.50<br>R^2=0.6684	22.04	L14	y=-3.1667x+97.5<br>R2=0.9757	15.00
L47	y=-3x+115.45<br>R^2=0.7072	21.82	L24	y=-3.5862x+102.07<br>R^2=0.9555	14.52
L57	y=-3.1356x+116.95<br>R^2=0.7610	21.35	L60	y=-2.900x+91.00<br>R^2=0.9397	14.14
L18	y=-3.1864x+117.29<br>R^2=0.7585	21.12	L26	y=-3.4815x+92.96<br>R^2=0.9321	12.34
L6	y=-3.0727x+114.73<br>R^2=0.7358	21.07	L23	y=-3.1316x+88.42<br>R^2=0.9116	12.27
L58	y=-3.2500x+118.39<br>R^2=0.7703	21.04	L3	y=-3.0833x+85.83<br>R^2=0.8692	11.62
L51	y=-3.375x+120.94<br>R^2=0.7745	21.02	L2	y=-3.4667x+89.78<br>R^2=0.8987	11.47
L17	y=-3.1833x+116.50<br>R^2=0.7707	20.89	L12	y=-3.2500x+84.17<br>R^2=0.8569	10.51
L35	y=-3.2034x+116.27<br>R^2=0.7852	20.69	L1	y=-3.3273x+84.91<br>R^2=0.8601	10.49
L56	y=-3.0167x+112.33<br>R^2=0.7695	20.66	L10	y=-3.2105x+82.64<br>R^2=0.8287	10.17
